# The Suppressive Effect of the Motor System on the Sensory System in Patients With Tourette Syndrome

**DOI:** 10.3389/fneur.2020.00855

**Published:** 2020-08-26

**Authors:** Ying Sun, Hua Wei, Yicong Lin, Yuping Wang

**Affiliations:** ^1^Department of Neurology, Xuanwu Hospital, Capital Medical University, Beijing, China; ^2^Beijing Key Laboratory of Neuromodulation, Beijing, China; ^3^Center of Epilepsy, Beijing Institute for Brain Disorders, Capital Medical University, Ministry of Science and Technology, Beijing, China

**Keywords:** Tourette syndrome, repetitive transcranial magnetic stimulation, somatosensory evoked potential, paired somatosensory evoked potential, motor cortex, sensory cortex

## Abstract

**Objective:** Tourette syndrome (TS) is a complicated sensorimotor disorder. Some patients with TS relieve their involuntary premonitory urges *via* tics. However, the effect of the motor system on the sensory system has not yet been elucidated. The purpose of the present study was to investigate changes in the excitability of the sensory cortex following repetitive transcranial magnetic stimulation (rTMS) of the motor cortex in patients with TS.

**Methods:** Twenty-nine patients with TS and 20 healthy, age-matched controls were enrolled in this study. All subjects were divided into four groups: patients with rTMS, patients with sham-rTMS, controls with rTMS, and controls with sham-rTMS. The clinical severity of tics was evaluated using the Yale Global Tic Severity Scale. Single somatosensory evoked potentials (SEPs) and paired SEPs were recorded by stimulating the median nerve at the wrist of all subjects. The resting motor threshold (RMT) was tested in each subject in the rTMS group. Afterwards, all four groups were administered rTMS (1 Hz, 90% RMT) or sham-rTMS for 200 s, followed by a 15-min rest. Finally, single SEPs and paired SEPs were repeated for each subject.

**Results:** No significant differences in RMT, the amplitudes of single SEPs, or the suppression of paired SEPs were observed between patients with TS and controls at baseline. After rTMS, a significant suppression of the peak-to-peak amplitude of the N20–P25 responses of single SEPs was observed in both controls (*p* = 0.049) and patients (*p* < 0.0001). The suppression of the N20–P25 peak-to-peak amplitude was more significant in patients than in controls (*p* = 0.039). A significant difference in the suppression of paired SEPs after rTMS was not observed between groups.

**Conclusions:** The more significant suppression of N20–P25 components of single SEPs with normal suppressed paired SEPs in patients with TS after 1-Hz rTMS of the motor cortex suggests that the suppressive effect of the motor system on the sensory system might originate from the motor-sensory cortical circuits rather than the sensory system itself.

## Introduction

Tourette syndrome (TS) is a complicated neuropsychiatric syndrome whose primary symptoms include chronic motor and vocal tics (1, 2). Tics are defined as quick, rapid, recurrent, non-rhythmic, brief movements, or vocalizations with a waxing and waning course (3). TS is considered a complicated sensorimotor syndrome rather than a purely motor disease. Some tics may be preceded by a premonitory sensation or urge (4–6), and tics may relieve the “involuntary” sensations in patients with TS. The pathophysiological mechanism of TS remains incompletely understood, but researchers generally agree that the cortical–striatal–thalamic–cortical circuits are likely to be dysfunctional in these patients (7, 8).

Previous studies on connectivity within sensory and motor circuits in patients with TS have shown reduced motor–motor inhibition [short interval intracortical inhibition (SICI)] and that sensory–motor inhibition [short-latency afferent inhibition (SAI)] reduced (9, 10) using transcranial magnetic stimulation (TMS), which supports clinical observations indicating a role for sensory symptoms in provoking tics.

In addition, internal sensory urges are often relieved by the tics in the same area in patients with TS (11). Therefore, we speculated that the motor system might exert some effects on the sensory system in patients with TS. An efficient approach to study the motor-sensory pathways is to combine repetitive transcranial magnetic stimulation (rTMS) and somatosensory evoked potentials (SEPs) to explore the interaction between input and output circuits. Enomoto et al. (12) observed decreased sensory cortical excitability in healthy volunteers after performing 1-Hz rTMS on the ipsilateral primary motor cortex. We investigated the changes in SEPs after delivering rTMS in the motor cortex of patients with TS to explore the effect of motor activation on the sensory system in patients with TS. We added a further experiment assessing the effects of rTMS on paired SEPs to determine whether the motor-sensory inhibitive effect was related to the sensory system itself. Some previous studies of paired SEPs (13, 14) showed suppression of SEPs in response to the second stimulus at short interstimulus intervals (ISIs) (20–100 ms). This method occasionally reveals changes in the excitability of the somatosensory cortex in patients who showed no conduction delays in conventional SEPs (15).

In the present study, we investigated the changes in the excitability of the sensory cortex, as indicated by SEPs and paired SEPs, occurring after the application of rTMS to the motor cortex, and we compared the effects between patients and healthy controls.

## Materials and Methods

### Participants

Twenty-nine patients with a diagnosis of TS without comorbidities (attention-deficit/hyperactivity disorder or obsessive-compulsive disorder) according to *Diagnostic and Statistical Manual of Mental Disorders*, fourth edition (DSM-IV) (16), and 20 healthy subjects were recruited for our study. All patients underwent a complete neurological examination, which revealed no additional abnormalities. None of the patients had a family history of neurological disorders. The results of neuroimaging, including cranial CT/MRI, were normal. On the day of the experiments, the severity of tics was rated using the Yale Global Tic Severity Scale (YGTSS) (17). None of the patients had taken medication for at least 2 weeks prior to the study. All patients were divided into two groups: patients with rTMS and patients with sham-rTMS. The control group consisted of 20 age-matched healthy subjects with a normal neurological examination and no history of neurological disorders. The controls were also divided into two groups: controls with rTMS and controls with sham-rTMS. All patients and controls were right-handed.

Written informed consent for the study and publication of study data was obtained from all participants and their guardians before inclusion. All procedures of the study received approval from the Xuanwu Hospital Ethics Committee and were conducted in accordance with the Declaration of Helsinki.

### Electromyography Recordings

Surface electromyograms (EMGs) of the right abductor pollicis brevis (APB) muscle were recorded using Ag–AgCl disc surface electrodes (9 mm in diameter) in a tendon-belly montage. The EMG signal was amplified and filtered (bandwidth of 20–10,000 Hz), displayed on a screen, converted with an analog-to-digital interface, and stored for further analysis.

### Transcranial Magnetic Stimulation

All subjects were seated in a comfortable reclining chair so that they were relaxed during the examinations, and their muscle tone was continuously monitored using audio-visual EMG. TMS was performed with a figure-eight coil (87-mm external diameter, peak magnetic field, 2.2 T) powered by a Magstim Super Rapid Stimulator (Magstim Company, Whitland, Dyfed, UK). This stimulator generated a magnetic pulse with a monophasic waveform and induced a current with posterior–anterior flow in the brain when the coil handle was positioned at an angle of 45° pointing backward. The direction of the electric current was approximately perpendicular to the central sulcus. The orientation of this induced electrical field is designed to produce trans-synaptic activation of the corticospinal neurons (18).

We located the hand motor area on the scalp of the left hemisphere. We moved the coil on the presumed area of the left hand motor cortex in 1-cm steps and delivered a single-pulse TMS to induce EMG responses (the number of stimuli delivered at each spot while searching for the hotspot was 2–4). The area that showed the largest EMG amplitude in the right APB muscle was defined as the hand motor cortex area. It was marked on the scalp with a red pen to ensure accurate repositioning of the coil throughout the examination. The stimulus intensity was presented as a percentage of the maximal stimulator output.

### Resting Motor Threshold

The resting motor threshold (RMT) was determined using the recommended method from the International Federation of Clinical Neurophysiology (IFCN) Committee, which was the intensity of stimulation eliciting at least five motor evoked potentials (MEPs) of 50 μV in 10 consecutive trials (19).

### Repetitive Transcranial Magnetic Stimulation and Sham Repetitive Transcranial Magnetic Stimulation

One train of 200 stimuli was administered to the hand motor area at a frequency of 1 Hz (12). The intensity was fixed at 90% RMT to exclude the disturbance of the afferent input provoked by an induced muscle twitch (20). Sham-rTMS was delivered through the same figure-of-eight coil, which was positioned 10 cm above the hand motor area (21) and fixed for the remainder of the experiment. The intensity was fixed at the minimal stimulator output (10%). During sham-rTMS, the coil was repetitively discharged, which was considered to produce an acoustic stimulation but no effective current flow in the cortex.

### Somatosensory Evoked Potentials

SEPs were recorded by stimulating the median nerve at the wrist using the cathode of the standard bar electrodes positioned proximally, to study changes in the excitability of the primary somatosensory cortical area.

### Single Somatosensory Evoked Potentials

SEPs were recorded in the left sensory area after stimulating the right median nerve at the wrist (0.2-ms square waves delivered by Neuropack M1) with a frequency of 1 Hz and an intensity just above the thumb twitch threshold. SEPs were recorded at frontal (F3, 5 cm anterior to C3 of the International 10-20 system) and parietal (C3′, 2 cm posterior to C3) electrodes with a reference electrode on the ipsilateral earlobe. At least 200 responses were averaged (bandpass, 5–3,000 Hz), with automatic rejection of samples with excessive EMG interference and stored for further analysis. We identified the following SEP components: the P14, N18, P22, and N30 potentials were recorded over the contralateral frontal region; and the P14, N20, and P25 potentials were recorded over the parietal region contralateral to the stimulation side. The latencies and the peak-to-peak amplitudes of the frontal potential (P14–N18, N18–P22, and P22–N30 components) and the parietal potential (P14–N20 and N20–P25 components) were measured (P14 and N18 do not represent cortical potentials). The SEP responses were recorded twice for each subject to verify the consistency of the potentials. The average SEPs were designated as single SEPs (S-SEPs).

### Paired Somatosensory Evoked Potentials

The paired stimulation SEPs showed deep inhibition at ISIs of 20–50 ms (13–15, 21, 22). We studied paired stimulation SEPs at an ISI of 40 ms. Paired stimuli (S1 and S2) of equal intensities were administered to the right median nerve. Each pair of stimuli was administered at a frequency of 1 Hz, and 200 responses were recorded and averaged. The duration and intensity of the stimuli and the recording parameters were the same as those used for single SEPs. The first stimulus was the conditioning stimulus, and the second stimulus was the test stimulus. SEPs evoked by the test stimulus (T-SEPs) were obtained by subtracting SEPs evoked by a single stimulus alone (S-SEPs) from SEPs elicited with paired stimuli (P-SEPs). We measured the amplitudes of each component in the subtracted SEP waveform and calculated the relative amplitude ratios of T-SEPs to the corresponding S-SEPs.

### Procedure

**Figure 1:**
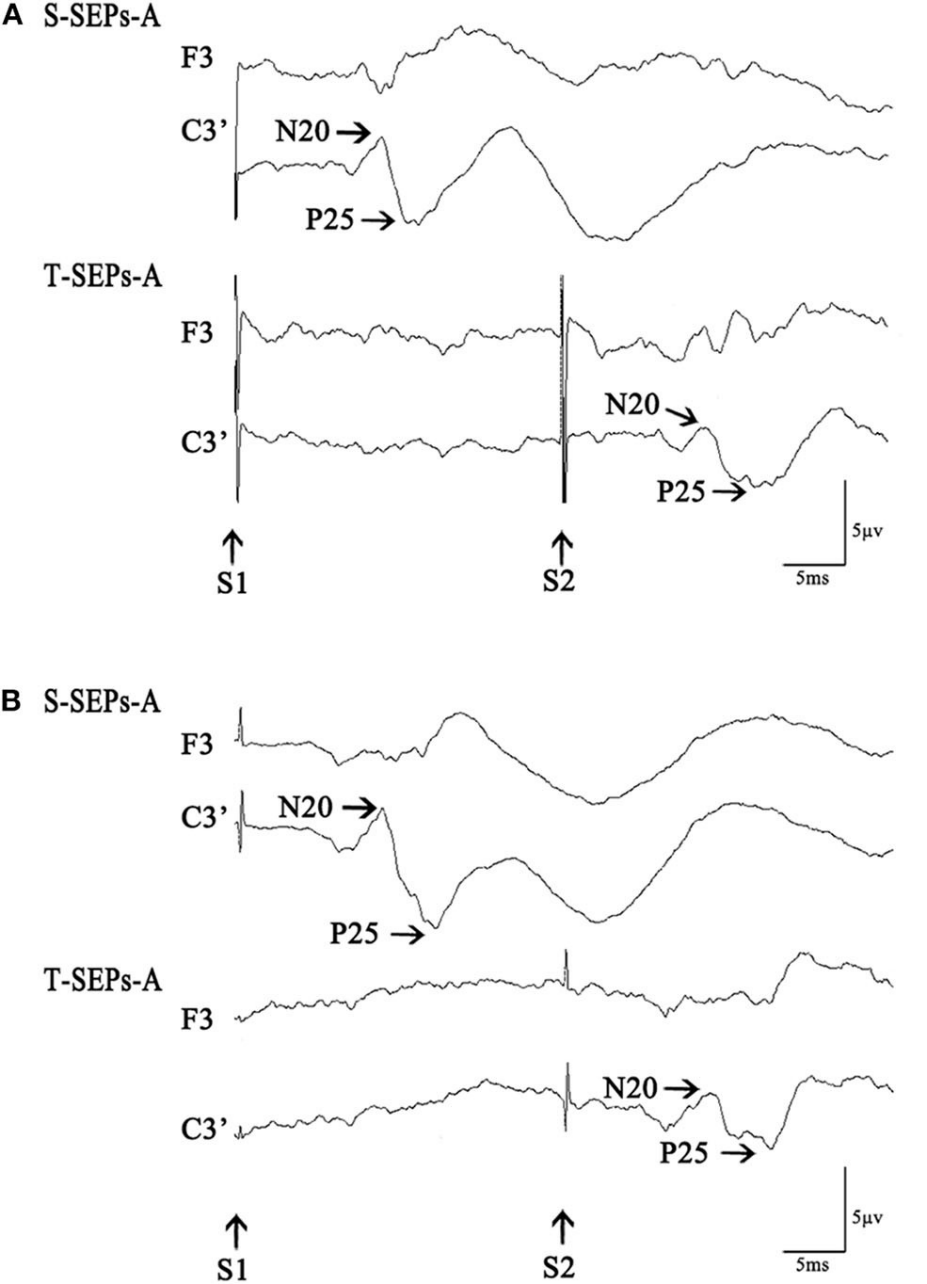
S-SEPs-A and T-SEPs-A in a control subject **(A)** and a Tourette syndrome (TS) patient **(B)**. S-SEPs-A are somatosensory evoked potentials by a single-pulse stimulus. T-SEPs-A are obtained by subtracting S-SEPs-A from somatosensory evoked potentials elicited by paired stimuli (P-SEPs-A) at an interstimulus interval (ISI) of 40 ms.

**Figure 2:**
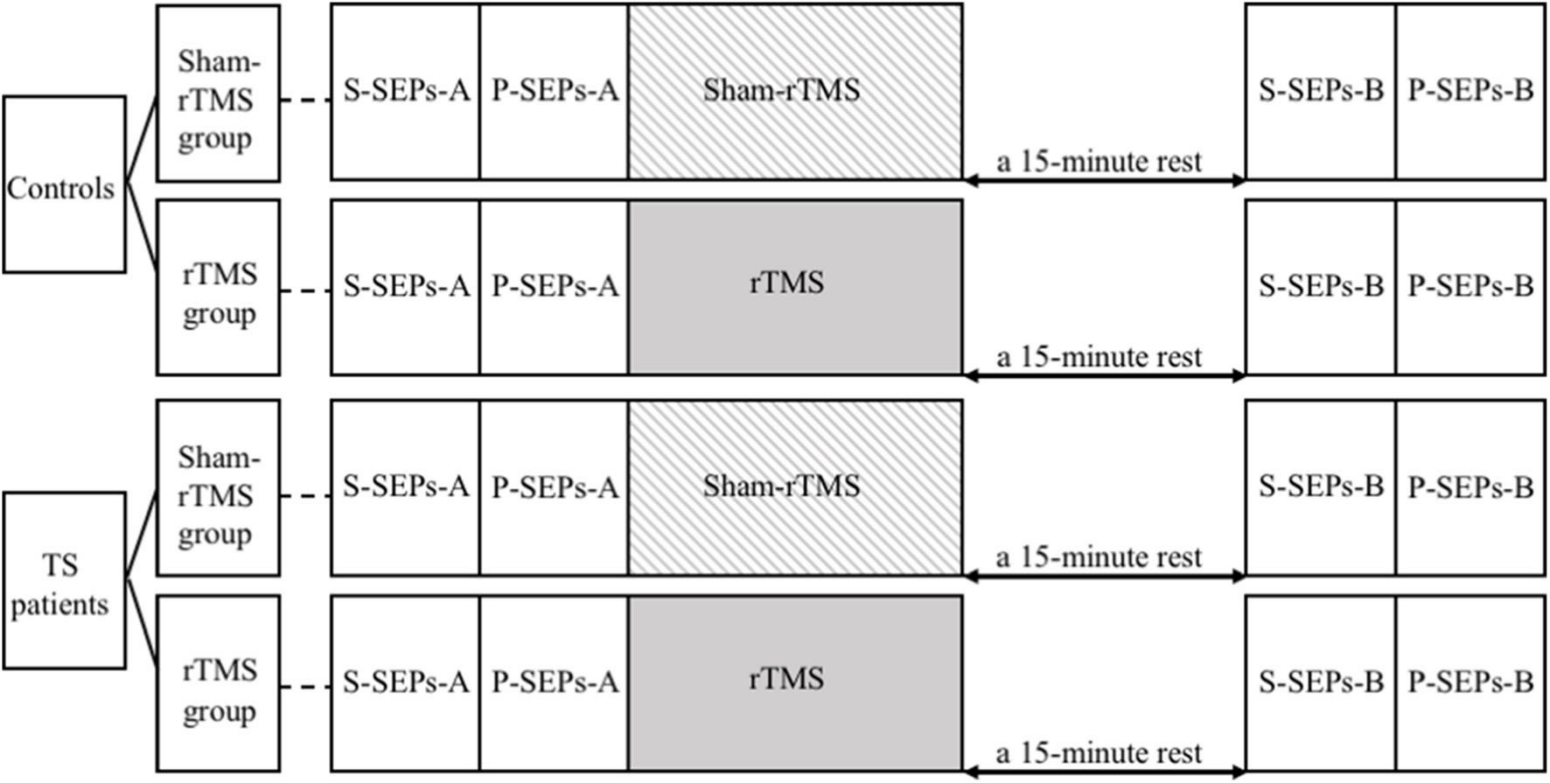
The study procedures. S-SEPs-A, single somatosensory evoked potentials (SEPs) recorded before repetitive transcranial magnetic stimulation (rTMS)/sham-rTMS; P-SEPs-A, paired SEPs recorded before rTMS/sham-rTMS; S-SEPs-B, single SEPs recorded after rTMS/sham-rTMS; P-SEPs-B, paired SEPs recorded after rTMS/sham-rTMS.

We recorded single SEPs (S-SEPs-A) and paired SEP (P-SEPs-A) for each subject (Figure 1). Then, the RMT was determined for each subject in the rTMS group. Afterwards, all four groups were administered rTMS/sham-rTMS for 200 s, followed by a 15-min rest. Finally, we examined and recorded another group of single SEPs (S-SEPs-B) and paired SEPs (P-SEPs-B) for each subject (Figure 2).

### Data Analysis

The statistical analysis was performed using SPSS 23.0 software. Statistical comparisons of the demographic data and clinical characteristics between the patients and the controls were performed using an independent-samples *t*-test. The same test was performed to compare the RMTs for patients and controls with rTMS, as well as the amplitudes and latencies of S-SEPs-A and paired-pulse ratios of T-SEPs-A/S-SEPs-A in the patients and the controls. The amplitudes of S-SEPs-B were expressed as a ratio of S-SEPs-A to minimize individual variability. S-SEPs-B were compared among the four groups by using one-way ANOVA and Bonferroni's *post hoc* analysis. The changes in T-SEPs are presented as ratios (T-SEPs-B/T-SEPs-A), and these ratios were compared among the four groups by using one-way ANOVA and Bonferroni's *post hoc* analysis. The results are reported as the means ± standards error (X ± SE). Results were considered statistically significant at a level of *p* < 0.05. The individuals who performed the analysis were blinded to the group assignments.

## Results

None of the subjects reported any adverse side effects during the course of the study.

### Demographic and Clinical Characteristics

The demographic and clinical characteristics of the subjects are summarized in Table 1. Mean age, mean body height, and YGTSS scores were not significantly different among the groups (mean age, *p* = 0.28; mean body height, *p* = 0.45; and YGTSS scores, *p* = 0.85).

**Table 1:** Demographic and clinical characteristics of the subjects.

	**Controls**		**Patients**	
**Variable**	**Sham-rTMS**	**rTMS**	**Sham-rTMS**	**rTMS**
Cases	10	10	10	19
Sex				
Male	8	5	8	16
Female	2	5	2	3
Mean age (years)	15.82 ± 0.70	17.85 ± 0.64	18.33 ± 1.48	18.17 ± 0.80
Mean body height (m)	1.72 ± 0.02	1.69 ± 0.03	1.66 ± 0.02	1.70 ± 0.02
YGTSS scores			38.80 ± 0.76	39.05 ± 0.83

*Mean age, mean body height, and YGTSS scores: mean ± standard error*.

*rTMS, repetitive transcranial magnetic stimulation; YGTSS, Yale Global Tic Severity Scale*.

### Resting Motor Threshold

The RMT was not significantly different between controls and patients with rTMS (controls, 53.2 ± 1.2%; patients, 57.2 ± 1.6%; *p* = 0.11), which indicated no significant differences in the intensity of rTMS between the groups.

### S-SEPs-A and T-SEPs-A Before Repetitive Transcranial Magnetic Stimulation

**Figure 3:**
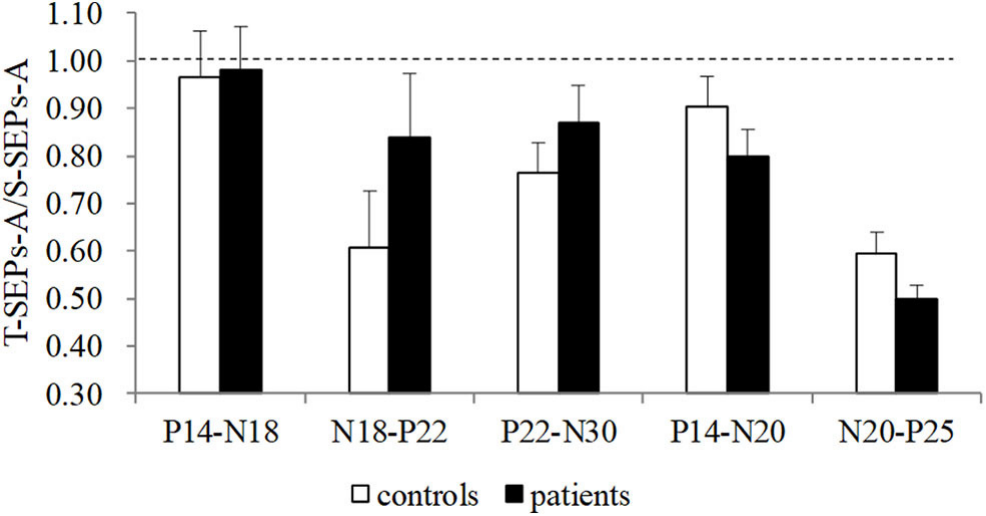
The amplitude ratios of T-SEPs-A/S-SEPs-A in controls and TS patients. A significant difference was not observed between controls and patients (*p* > 0.05). SEP, somatosensory evoked potential; T-SEP, test SEP; S-SEP, single SEP.

No significant differences in the latencies and amplitudes of S-SEPs-A were observed between controls and patients. Mean latencies and amplitudes of S-SEPs-A are shown in Tables 2, 3. In each group, the amplitude ratios of T-SEPs-A <1, but the amplitude ratios of T-SEPs-A were not significantly different between controls and patients (P14–N18, *p* = 0.90; N18–P22, *p* = 0.23; P22–N30, *p* = 0.35; P14–N20, *p* = 0.23; and N20–P25, *p* = 0.07; Figure 3).

**Table 2:** Mean latencies (ms) of S-SEP-A components in controls and TS patients.

**Site**	**Components**	**Controls (ms)**	**Patients (ms)**	***p*-value**
F3	P14	13.77 ± 0.26	13.52 ± 0.19	0.44
	N18	16.54 ± 0.26	16.49 ± 0.25	0.90
	P22	19.26 ± 0.31	19.32 ± 0.28	0.89
	N30	29.51 ± 0.54	29.12 ± 0.47	0.59
C3′	P14	14.19 ± 0.25	13.64 ± 0.19	0.08
	N20	18.55 ± 0.22	18.18 ± 0.15	0.16
	P25	23.41 ± 0.57	23.26 ± 0.36	0.83

*The data were expressed as the mean ± standard error*.

*S-SEP, single somatosensory evoked potential; TS, Tourette syndrome*.

**Table 3:** Mean amplitudes (μV) of S-SEP-A components in controls and TS patients.

**Site**	**Components**	**Controls (μV)**	**Patients (μV)**	***p*-value**
F3	P14–N18	1.39 ± 0.13	1.44 ± 0.11	0.77
	N18–P22	1.39 ± 0.17	1.66 ± 0.23	0.39
	P22–N30	4.09 ± 0.40	5.06 ± 0.47	0.15
C3′	P14–N20	3.35 ± 0.30	3.49 ± 0.27	0.73
	N20–P25	5.31 ± 0.57	6.34 ± 0.81	0.34

*The data were expressed as the mean ± standard error*.

*S-SEP, single somatosensory evoked potential; TS, Tourette syndrome*.

### S-SEPs-B and T-SEPs-B After Repetitive Transcranial Magnetic Stimulation

**Figure 4:**
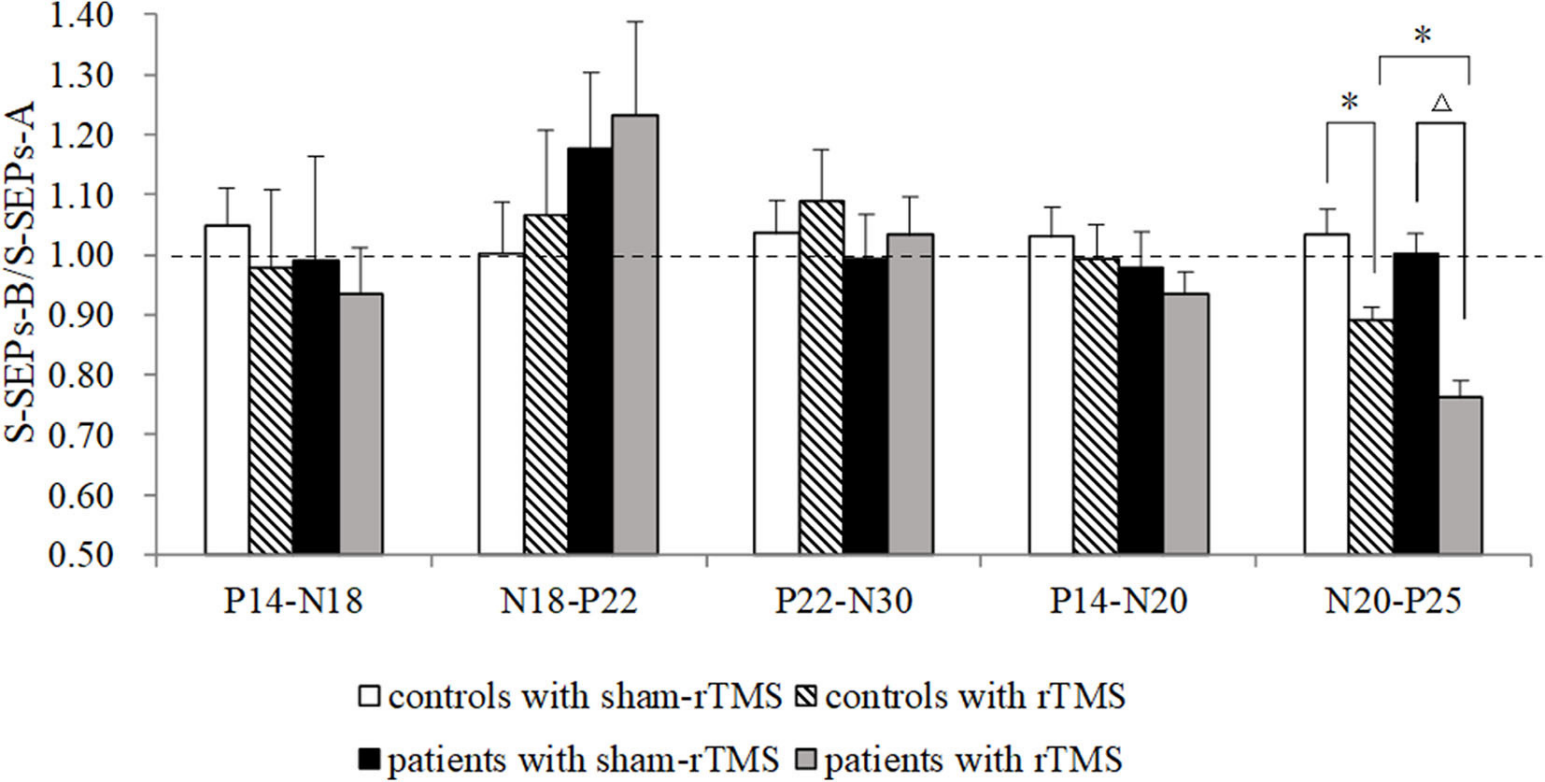
Effects of repetitive transcranial magnetic stimulation (rTMS) on the primary motor cortex on single somatosensory evoked potentials (SEPs) in controls and Tourette syndrome (TS) patients. A marked decrease was observed in the amplitude of N20–P25 in controls and patients after rTMS. The N20–P25 component of S-SEP-B in patients was significantly different from controls. The amplitudes of P14–N18, N18–P22, P22–N30, and P14–N20 were not significantly different among the groups. *p < 0.05 and ^Δ^*p* < 0.01.

The amplitude ratios of S-SEPs-B in the four groups showed significant differences (Figure 4). Notably, rTMS led to a significant decrease in the peak-to-peak amplitude of N20–P25 in controls (*p* = 0.049) and patients (*p* < 0.0001) compared with the respective sham-rTMS groups. The decrease was more significant in patients with rTMS than in controls with rTMS (*p* = 0.039). The amplitudes of P14–N18, N18–P22, P22–N30, and P14–N20 were not significantly different among the groups (P14–N18, *p* = 0.90; N18–P22, *p* = 0.67; P22–N30, *p* = 0.85; and P14–N20, *p* = 0.52). Finally, the ratios of T-SEPs-B/T-SEPs-A showed no significant differences among the groups (Table 4).

**Table 4:** The ratios of T-SEP-B/T-SEP-A in controls and TS patients.

**Site**	**Component**	**Controls with sham-rTMS**	**Controls with rTMS**	**Patients with sham-rTMS**	**Patients with rTMS**	***p*-value**
F3	P14–N18	1.36 ± 0.29	1.31 ± 0.23	2.56 ± 0.96	1.31 ± 0.17	0.17
	N18–P22	2.44 ± 1.42	2.09 ± 0.53	0.76 ± 0.20	1.26 ± 0.21	0.31
	P22–N30	1.29 ± 0.29	1.40 ± 0.34	0.87 ± 0.05	1.22 ± 0.16	0.47
C3′	P14–N20	1.00 ± 0.23	1.05 ± 0.15	2.23 ± 0.99	1.22 ± 0.11	0.22
	N20–P25	1.09 ± 0.17	1.17 ± 0.16	1.37 ± 0.21	1.51 ± 0.12	0.21

*The data were expressed as the mean ± standard error*.

*T-SEP, test somatosensory evoked potential; TS, Tourette syndrome; rTMS, repetitive transcranial magnetic stimulation*.

## Discussion

In the present study, we evaluated the changes in S-SEPs and T-SEPs after applying 1-Hz rTMS on the motor cortex. Significant suppression of the peak-to-peak amplitude of the N20–P25 responses of S-SEPs was observed in both patients and controls. The suppression of the N20–P25 peak-to-peak amplitude was more significant in patients than in controls. No significant difference in the suppression of the P-SEPs was observed after rTMS between groups.

The primary symptom of TS is involuntary tics, and thus many previous electrophysiological studies have focused on motor cortical excitability. Many studies have observed an increase in motor cortex excitability in patients with TS after TMS of the motor cortex (9, 10, 23). The cortical silent period (CSP) and SICI are postulated to be specifically related to the motor cortex inhibitory mechanism, which reflects motor–motor inhibition. Ziemann et al. (9) observed CSP shortening and a defective reduction of SICI in patients with TS. The probable interpretation was that tics in patients with TS originated from a primarily subcortical disorder that affected the motor cortex through disinhibited afferent signals, impaired inhibition at the level of the motor cortex directly, or both. Brain imaging studies (24) have further revealed that the hyperexcitability within the primary motor cortex (M1) observed in patients with TS is likely caused by increased functional interactions between the supplementary motor area (SMA) and M1. These results revealed the interactions within the motor system of patients with TS.

According to clinical observations (4–6, 11), TS is a sensorimotor disorder rather than a purely motor disease, and the sensory system also participates in the pathophysiological mechanisms. One proven pathway is SAI, in which a transient sensory input leads to rapid and short-term inhibition of the motor cortex (25). As shown in some studies (10, 23), SAI is suppressed in patients with TS, indicating that impaired intracortical inhibition might not be limited to the motor cortex and might also involve sensory input and motor output circuits. Given the possible role of sensory inputs in triggering tics, reduced SAI may be a direct physiological consequence of decreased suppression from sensory inputs to motor outputs in patients with TS.

Previous reports have presented pathophysiological hypotheses for tics in patients with TS on the basis of potential abnormalities in the motor–motor inhibition and sensory–motor pathways. In patients with TS, internal sensory urges are often relieved by a tic in the area. Based on this clinical phenomenon, the motor system may exert effects on the sensory system in patients with TS. However, the mechanism underlying the motor cortex-mediated activation of the sensory system in patients with TS remains unknown.

### Baseline (Before Repetitive Transcranial Magnetic Stimulation) Excitability of Sensory System in Patients With Tourette Syndrome

SEPs have been used to detect different levels of excitability in the sensory system. Consistent with the results of previous electrophysiology studies (26, 27), we observed normal latencies and the amplitudes of the frontal components (P14–N18, N18–P22, and P22–N30) and the parietal components (P14–N20 and N20–P25) in patients with TS. However, the normal SEPs of patients with TS only confirm the integrity of the sensory pathways. Processing in the sensory cortex remains unclear. The paired stimulation SEP technique has been used to study the recovery function of the somatosensory cortex in patients with some neurological disorders (15, 22, 28, 29). The paired SEPs showed deep inhibition at ISIs of 30–50 ms (13–15, 21, 22). In our experiment, we studied the suppression at an ISI of 40 ms in the paired SEPs. The amplitudes of the frontal components (P14–N18, N18–P22, and P22–N30) and the parietal components (P14–N20 and N20–P25) were suppressed in both controls and patients with TS. Thus, sensory activation probably suppressed the subsequent sensory system processing. In addition, we did not observe a significant difference in the suppression of paired SEPs between the controls and patients, suggesting the lack of a significant difference in the effect of sensory cortical activation on the sensory information input between controls and patients with TS.

### Effects of Repetitive Transcranial Magnetic Stimulation on the Motor Cortex to the Excitability of Sensory System in Patients With Tourette Syndrome

TMS has been used to alter the excitability of the cortex and to help researchers investigate the integration of sensory afferents with motor output (25). Because the modulatory effects of rTMS not only are limited to the stimulated cortex but also mediate functional changes in interconnected cortical areas, rTMS would be a suitable tool to investigate plasticity within the distributed functional network. We studied the changes in S-SEPs and P-SEPs after delivering rTMS on the motor cortex to reveal the potential effect of the motor system on the sensory system in patients with TS.

The frequency of rTMS has been proven to be an important influencing factor in determining whether lasting modulatory effects on the cortex are predominantly facilitatory or inhibitory (30, 31). Low-frequency rTMS reduces cortical excitability in the local cortical region and in the functionally linked cortical regions. According to Enomoto et al. (12), ipsilateral N20–P25 peak-to-peak amplitudes are suppressed after the administration of 1-Hz rTMS on the motor cortex, and rTMS applied on the premotor cortex or sensory cortex or sham stimulation does not exert a suppressive effect on SEPs in healthy subjects. Consistent with previous studies (10, 23), the RMT did not differ between patients with TS and controls in our study. However, the parietal N20–P25 amplitudes were significantly suppressed after 1-Hz rTMS on the motor cortex in controls and patients. As shown in previous studies, the parietal N20 component originates from the anterior bank of the post-central gyrus (area 3b) and reflects the activation of the sensory cortex by thalamocortical fibers (12, 32). P25 is generated at the level of the sensory cortex (33, 34). Therefore, suppression occurred in the sensory cortex after 1-Hz rTMS on the primary motor cortex in the present study. The suppression of SEPs in the ipsilateral sensory cortex was more likely to be produced by cortico-cortical effects. Furthermore, the parietal N20–P25 amplitudes were suppressed to a markedly greater extent in patients than in controls with rTMS. Thus, the suppressive effect of the motor cortex on the sensory cortex was more significant in patients with TS than in the controls, and the suppression was produced by cortico-cortical effects from the motor cortex to the sensory cortex.

We have not yet clearly determined whether these effects are related to an active process of inhibition of the sensory cortex. We further investigated the changes in paired SEPs after 1-Hz rTMS of the motor cortex. A gamma-aminobutyric acid (GABA) receptor-mediated mechanism that reduces transmitter release in the cerebral cortex might play an important role in the mechanism of paired-pulse inhibition (35–37). After subthreshold 1-Hz rTMS to the primary motor cortex was applied, no change was observed in the suppression of paired SEP, and a significant difference was not observed between patients and controls. This finding probably indicated that subthreshold low-frequency rTMS (1 Hz) on the primary motor cortex did not induce a meaningful change in inhibitory circuits in the sensory system. Because patients with TS had more significantly suppressed S-SEPs and ordinary level of suppression of P-SEPs after rTMS, the motor-sensory cortex circuits exerted the greater inhibitory effect on the sensory system of patients with TS, rather than the sensory system itself.

This study has a few limitations. First, sham-rTMS was delivered through the coil, which was fixed 10 cm above the hand motor area. Therefore, the cutaneous sensation on the scalp was also absent, as compared with that of the rTMS group. Second, single SEPs and paired SEPs were recorded only 15 min after the rTMS/sham-rTMS, and thus the potential longer-term effects of rTMS remain unclear and should be explored in the future. Third, this study had a relatively limited sample size. Future studies with larger sample sizes should be conducted to confirm the conclusions.

## Conclusion

We investigated the changes in single SEPs and paired SEPs after delivering 1-Hz rTMS on the motor cortex to study the effect of motor activation on the sensory system in patients with TS. In our experiment, the motor system exerted a more significant effect on suppressing the sensory system in patients with TS, and this suppression might originate from the motor-sensory cortex circuits rather than the sensory system itself.

## Data Availability Statement

The raw data supporting the conclusions of this article will be made available by the authors, without undue reservation.

## Ethics Statement

The studies involving human participants were reviewed and approved by Xuanwu Hospital Ethics Committee. Written informed consent to participate in this study was provided by the participants' legal guardian/next of kin.

## Author Contributions

YS, HW, and YW collected the patients. YS acquired the clinical and SEPs data and wrote the manuscript. YW designed the study. YL analyzed and interpreted the SEPs data. YW and YL critically revised the manuscript. All authors contributed to the article and approved the submitted version.

## Conflict of Interest

The authors declare that the research was conducted in the absence of any commercial or financial relationships that could be construed as a potential conflict of interest.
